# Gestational age assessment in malaria pregnancy cohorts: a prospective ultrasound demonstration project in Malawi

**DOI:** 10.1186/1475-2875-12-183

**Published:** 2013-06-04

**Authors:** Blair J Wylie, Linda Kalilani-Phiri, Mwayi Madanitsa, Gladys Membe, Osward Nyirenda, Patricia Mawindo, Redson Kuyenda, Albert Malenga, Abbey Masonbrink, Bonus Makanani, Phillip Thesing, Miriam K Laufer

**Affiliations:** 1 Division of Maternal-Fetal Medicine, Department of Obstetrics and Gynecology, Massachusetts General Hospital, 55 Fruit Street, Boston, Massachusetts, Boston, Massachusetts, USA 02114; 2 University of Malawi College of Medicine, Blantyre, Malawi; 3 The Blantyre Malaria Project, Queen Elizabeth Central Hospital, Chipatala Avenue, PO Box 32256, Blantyre 3, Blantyre, Malawi; 4 New York Department of Health, New York, New York, USA; 5 City of Cincinnati Health Department, Cincinnati, Ohio, USA; 6 Center for Vaccine Development, School of Medicine, University of Maryland, Baltimore, Maryland, USA

**Keywords:** Malaria in pregnancy, Ultrasound, Foetal biometry, Preterm delivery, Small for gestational age, Ballard examination

## Abstract

**Background:**

Malaria during pregnancy is associated with an increased risk for low birth weight (<2500 grams). Distinguishing infants that are born premature (< 37 weeks) from those that are growth-restricted (less than the 10th percentile at birth) requires accurate assessment of gestational age. Where ultrasound is accessible, sonographic confirmation of gestational age is more accurate than menstrual dating. The goal was to pilot the feasibility and utility of adding ultrasound to an observational pregnancy malaria cohort.

**Methods:**

In July 2009, research staff (three mid-level clinical providers, one nurse) from The Blantyre Malaria Project underwent an intensive one-week ultrasound training to perform foetal biometry. Following an additional four months of practice and remote image review, subjects from an ongoing cohort were recruited for ultrasound to determine gestational age. Gestational age at delivery established by ultrasound was compared with postnatal gestational age assessment (Ballard examination).

**Results:**

One hundred and seventy-eight women were enrolled. The majority of images were of good quality (94.3%, 509/540) although a learning curve was apparent with 17.5% (24/135) images of unacceptable quality in the first 25% of scans. Ultrasound was used to date 13% of the pregnancies when menstrual dates were unknown and changed the estimated gestational age for an additional 25%. There was poor agreement between the gestational age at delivery as established by the ultrasound protocol compared to that determined by the Ballard examination (bias 0.8 weeks, limits of agreement -3.5 weeks to 5.1 weeks). The distribution of gestational ages by Ballard suggested a clustering of gestational age around the mean with 87% of the values falling between 39 and 41 weeks. The distribution of gestational age by ultrasound confirmed menstrual dates was more typical. Using ultrasound confirmed dates as the gold standard, 78.5% of preterm infants were misclassified as term and 26.8% of small-for gestational age infants misclassified as appropriately grown by Ballard.

**Conclusion:**

Ultrasound should be strongly considered in prospective malaria studies with obstetric endpoints to confirm gestational age and avoid misclassification of infants as premature or growth-restricted. The use of ultrasound does require a significant investment of time to maintain quality image acquisition.

## Background

Malaria during pregnancy is associated with an increased risk for low birth weight, defined as weighing less than 2500 grams at the time of birth. Distinguishing those infants that are born early from those that are born small requires accurate assessment of gestational age. This distinction is crucial as the mechanisms and potential therapeutic interventions underlying premature labour and foetal growth restriction are distinct. Both processes have been implicated as consequences of pregnancy-associated malaria although growth restriction is thought to be more common than preterm labour in endemic areas [1]. The relative contribution of each, as well as appropriately targeted therapies, will remain unclear unless affected pregnancies are accurately dated.

Where ultrasound is routinely accessible, antenatal sonographic confirmation of gestational age is more accurate than menstrual dating alone when performed in the first half of gestation [2,3]. Other potential benefits of ultrasound in the research setting include demonstration of a live intrauterine pregnancy, determination of the number of foetuses being carried, and potentially increased satisfaction of the participating mother. Gestational age can be quite challenging to measure, even more so in resource-limited settings. In a review of pregnancy malaria studies reporting birth weight from 1966 to 2009, only 77% (33/43) of the publications even described their method of gestational age assessment [4]. Ultrasound was used, either alone or in combination with another gestational age assessment technique, in only 15% (5/33) of the studies with gestational age methods reported [5-9]. Notably, there has been a large increase in malaria pregnancy publications in the last decade [10] and the most recent publications are increasingly incorporating ultrasound into pregnancy dating [11-14].

In the review introduced above [4], the remainder of the study protocols dated pregnancies either before birth using menstrual dates, symphysis-fundal height, or a combination, or after birth with postnatal newborn maturity assessments such as the Dubowitz [15] or the Ballard [16]. The published accuracy of the new Ballard examination has been reported to confirm gestational age within a range of two weeks even for premature infants [16-18]. While validity studies were performed in populations around the globe (US, Japan, India) it is important to note that they were performed in tertiary institutions and in some cases with neonatologists performing the examinations. Whether similar results can be achieved in resource limited settings using non-physician staff remains unclear. Interestingly, one study suggests that the Ballard examination may systematically overestimate the gestational age of black infants in a United States hospital [19]. The implication for African newborns is uncertain. One additional downside of postnatal assessment of gestational age is that it cannot be used in the setting of a stillbirth as a live birth is required.

The overarching objective of this study was to pilot the addition of ultrasound to an observational malaria cohort for the purpose of gestational age assessment. The primary aim was to determine the feasibility of training midlevel non-obstetric clinicians to perform foetal biometry in a research setting. The secondary aim was to characterize the utility of adding ultrasound assessment of gestational age to a cohort of pregnant women by comparing antenatal ultrasound with postnatal Ballard assessment. Finally, it was hoped that the summary of findings might serve as a refresher on the concepts of gestational age assessment for non-obstetricians conducting malaria research in pregnant women.

## Methods

This ultrasound demonstration project was conducted as a substudy within a larger observational cohort study of pregnant women. The primary objective of the parent study was to evaluate the molecular epidemiology of malaria during pregnancy. Study activities were carried out at Ndirande Antenatal Care Clinic in Blantyre, Malawi. Four research staff, one registered nurse and three midlevel clinicians, underwent a one-week intensive ultrasound training during July 2009 in Blantyre led by the study perinatologist (BJW). Instruction was focused on sonographic assessment of gestational age by foetal biometry. Didactic instruction was followed by observed hands-on practice. Immediate feedback was provided when suboptimal images were taken and corrections made to the imaging plane and/or caliper placement. A portable SonoSite™ S180 machine (SonoSite, Inc, Bothell, Washington, USA) was used to obtain ultrasound images and was donated to the Blantyre Malaria Project by the Vincent Department of Obstetrics and Gynecology at Massachusetts General Hospital with a matching grant from the SoundCaring Program (Sonosite, Inc, Bothell, Washington, USA).

In the four months that followed the initial training, the four trained research staff continued practicing image acquisition using pregnant volunteers. De-identified images were then transmitted via the internet for critique by the study perinatologist. Following this additional four months of remote training, the formal ultrasound demonstration project commenced. Subjects were recruited from the ongoing observational cohort of pregnant women and provided informed consent in the native language of Chichewa for study procedures including ultrasound image acquisition of their foetus.

### Ultrasound protocol

To assess gestational age by ultrasound, the trained research staff worked in pairs to obtain at least two images of the three biometric parameters (biparietal diameter, femur length, and abdominal circumference). Sonographers were not blinded to the last menstrual period of the subject. If a multiple gestation or intrauterine foetal demise was identified at the time of the scan, the subject was excluded. An absolute value for each biometric measurement was recorded as was the corresponding gestational age generated by the SonoSite™ package software pre-programmed with specific nomograms for the biparietal diameter [20], femur length [20], and abdominal circumference [21]. The images were reviewed remotely by the study perinatologist and incorrectly obtained or measured images were excluded from the gestational age calculation using accepted standards for these measurements [22]. The gestational age was averaged for each parameter. If neither image was acceptable, this biometric parameter was deemed unsalvageable. The means of each biometric parameter were then averaged to generate an overall gestational age by ultrasound. A corresponding ultrasound estimated date of delivery (EDD) was determined using an electronic wheel available as an application for download on smart phones (Perfect OB Wheel, Dr. Evan Schoenberg) [23]. For foetuses measuring less than 20 weeks gestation, the abdominal circumference was not used in the estimation of the ultrasound EDD. No ultrasound EDD was calculated if more than one biometric parameter was unsalvageable. Of note, the ultrasound gestational age assessment was not used in the observational cohort as not all subjects in the parent trial underwent ultrasound examination.

### Gestational age protocol

A menstrual EDD was calculated using the self-reported first day of the last menstrual period and recorded as unknown if the subject was unaware of her last menstrual period. Women who were aware of the month of their last menstrual period but not the day were considered to have an unknown last menstrual period. Literacy among Malawian women is estimated to be 68% [24].

The menstrual EDD and the ultrasound EDD were then compared and a best obstetric estimate of the EDD assigned according to the protocol criteria outlined in Table 1. For pregnancies less than 20 weeks by ultrasound measurements, the ultrasound EDD became the best obstetric estimate of the EDD if there was a greater than seven-day discrepancy. For those measured between 20 and 28 weeks, a discrepancy of greater than 14 days resulted in re-dating the pregnancy by the ultrasound. In pregnancies measured beyond 28 weeks, re-dating occurred only if measurements were greater than 21 days different from menstrual dates given the limited ability of ultrasound to confirm gestational age in the third trimester when growth of individual foetuses can diverge. This method for assigning a best obstetric estimate of gestational age is referred to throughout this manuscript variable as ultrasound confirmed menstrual dates, ultrasound confirmed dates or simply the best obstetric estimate.

**Table 1:** **Protocol for re**-**dating the pregnancy by ultrasound**

**Biometric measurements**	**Gestational age by u/s**	**Accuracy**	**Criteria for re-dating**
BPD and FL	< 20 weeks	± 7 days	Pregnancy re-dated by u/s if measurements > 7 days different from menstrual dates
BPD, FL, & AC	20 to 28 weeks	± 14 days	Pregnancy re-dated by u/s if measurements > 14 days different from menstrual dates
BPD, FL, & AC	> 28 weeks	± 21 days	Pregnancy re-dated by u/s if measurements > 21 days different from menstrual dates

*u/s *ultrasound, *BPD *biparietal diameter, *FL *femur length, *AC *abdominal circumference.

Multiple studies support an accuracy of one week or less for sonographic measurements obtained at less than 20 weeks [25-27]. As gestation advances, variations in biometric measurements increasingly represent variability in foetal size rather than gestational age. Two weeks was chosen as the cutoff for re-dating pregnancies between 20 and 28 weeks and three weeks as the cutoff for re-dating pregnancies beyond 28 weeks as these represent accepted estimates of the variability (± 2 standard deviations) of ultrasound biometric measurements at these gestational ages [22]. Given that many women underwent their dating ultrasound examination beyond 20 weeks in this cohort, the EDD generated from ultrasound measurements alone was not used but rather the measurements were incorporated into a verification, or rejection, of menstrual dates. Only in cases when ultrasound measurements were discrepant from the menstrual dates or when the menstrual dates were unknown was the ultrasound EDD used.

### Delivery procedures and definitions

Following delivery, the infant was weighed using a digital scale and a postnatal new Ballard examination was performed within 36 hours of delivery by one of the four research nurses or two clinical officers. Examiners were not blinded to the last menstrual period of the subject. Both neurologic and external features were scored to generate a total Ballard score. The total score was correlated with gestational age using the published Ballard maturity-rating tables (Additional file 1). Weeks of gestation were defined as completed weeks without rounding of days. For example, pregnancies estimated anywhere from 27 weeks 0 days to 27 weeks 6 days would be considered 27 weeks gestation. A term delivery was defined as greater than 37 weeks on the day of delivery by the best obstetric estimate of gestational age; a preterm delivery was defined as a delivery occurring at less than 37 weeks by the best obstetric estimate. Infants were considered small-for-gestational age if their birth weight was less than the 10th percentile for gestational age by creating an East Africa specific curve [28]. Prior to launch of the study, research staff responsible for conducting the Ballard examinations were trained in its performance by the study physicians. A single brief refresher training course was held in the middle of the study period. Occasional spot checks of research staff were conducted by the study physicians but there was no formal quality control mechanism in place.

### Data analysis

The proportions of acceptable images for each biometric parameter were determined and were then compared by time since the initial ultrasound training. The utility of ultrasound in dating pregnancies with unknown last menstrual periods and the prevalence of discrepant menstrual and ultrasound dates were described. The agreement between the gestational age at delivery determined by the best obstetric estimate and that determined by postnatal Ballard examination was evaluated with the aid of a Bland-Altman plot. The distributions of gestational age for these two methods of gestational age assessment were compared visually and the medians compared using the Mann–Whitney test. Statistical calculations were performed using Microsoft Excel, 2003.

### Ethical considerations

The study protocol was approved by the University of Malawi College of Medicine Research and Ethics Committee in Blantyre, Malawi, the University of Maryland Institutional Review Board in Baltimore, Maryland, and the Partners Healthcare Institutional Review Board at Massachusetts General Hospital in Boston, Massachusetts.

## Results

One hundred and eighty women were recruited from the parent trial and enrolled into the ultrasound demonstration project. Of these, one set of twins was discovered at the time of the ultrasound scan and excluded from further analysis. Additionally, images from one additional subject were unsuccessfully transferred electronically and subsequently lost. This left 178 subjects for analysis.

### Quality of image acquisition

**Figure 1:**
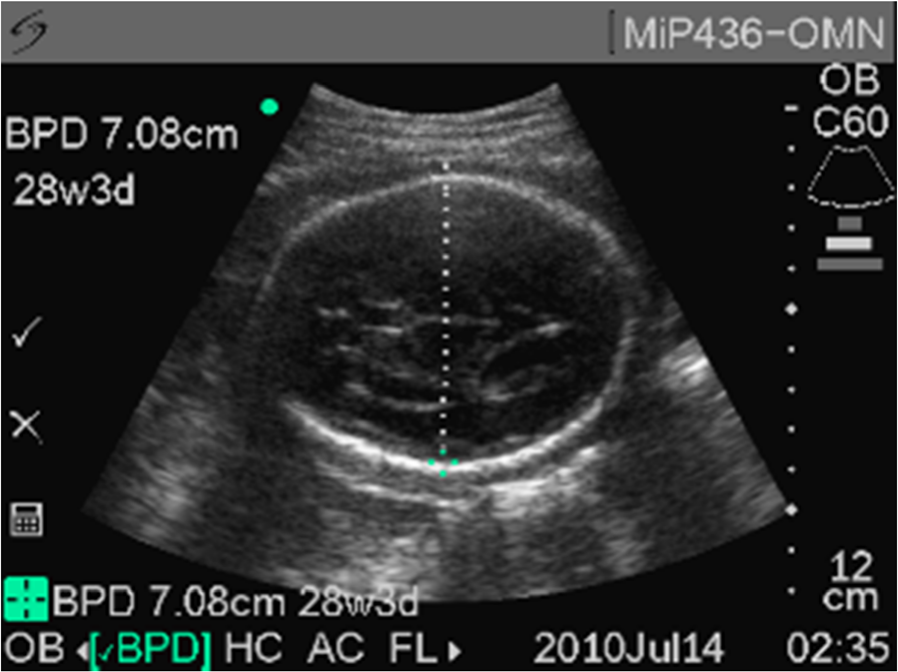
Representative sample image of biparietal diameter measurement.

**Figure 2:**
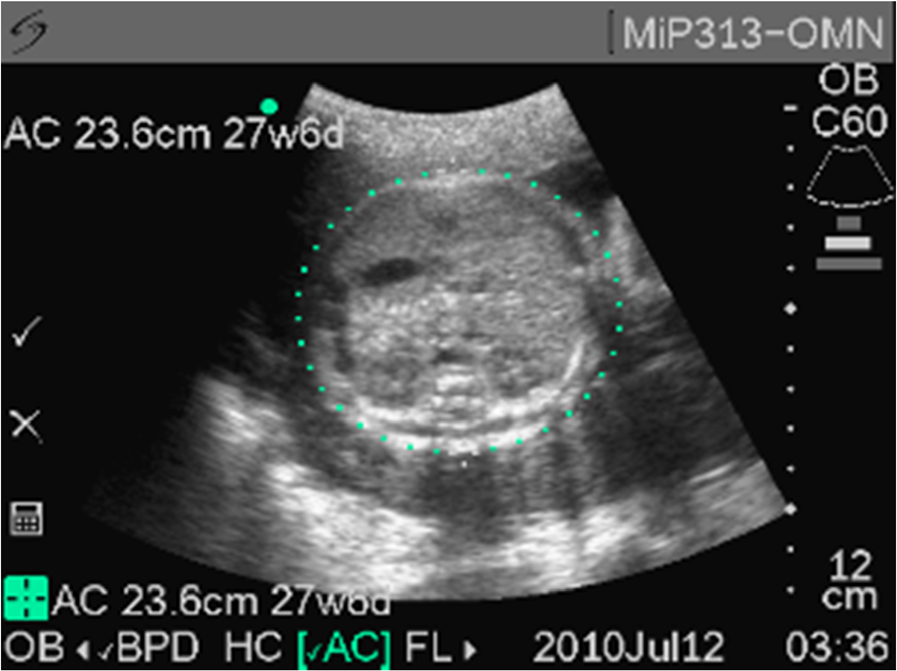
Representative sample image of abdominal circumference.

**Figure 3:**
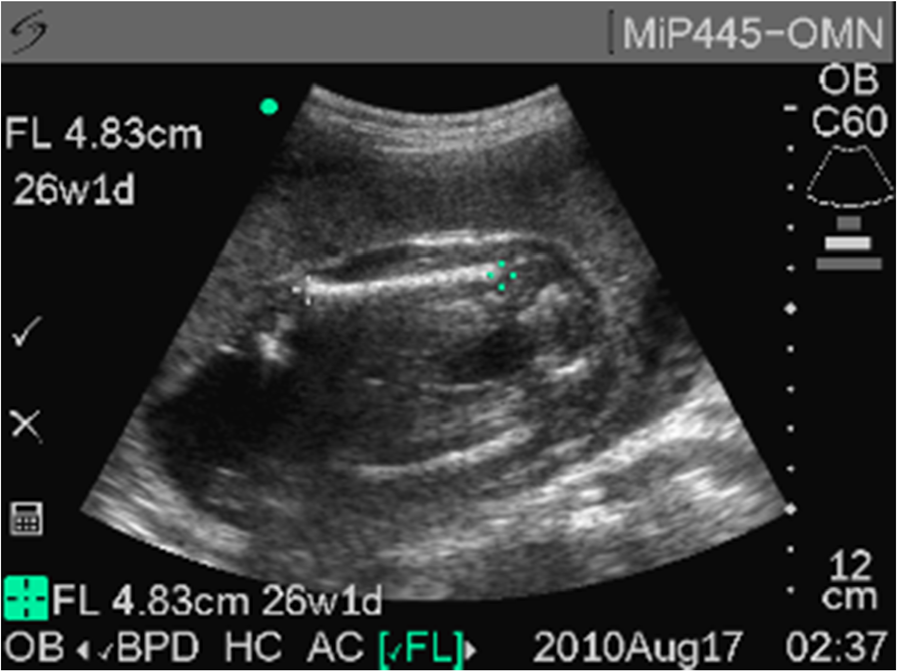
Representative sample image of femur length.

The vast majority of images were obtained properly with a biometric parameter deemed unsalvageable (neither image acceptable) in only 5.7% (31/540) of the biometric measurements attempted. Representative acceptable images are demonstrated in Figures 1, 2 and 3. In the first 45 subjects, 17.7% (24/135) of biometric assessments were unsalvageable compared to 1.7% (7/405) of images in the remaining 75% of subjects. This suggests that after an initial period of intense training and scrutiny image acquisition was consistently of good quality. In only one subject was more than one biometric parameter unsalvageable; this was the only case where an ultrasound EDD could not be estimated. Biparietal diameter and femur length measurements were deemed unsalvageable in only 5.1% (9/178) of subjects. While the abdominal circumference was more likely to be incorrectly estimated, the measurement was able to be estimated in 92.6% (165/178) of subjects.

### Timing of image acquisition

Only 16.3% (29 of 178 subjects) were imaged prior to 20 weeks. Most subjects (61.8%, 110/178) were enrolled and imaged between weeks 20 and 27. As this was in part an ultrasound demonstration project designed to improve the staff’s scanning skills throughout the gestational duration spectrum, pregnancies in the third trimester were not excluded. Thirty-eight subjects (21.3%) were imaged after 28 weeks. The one subject whose images were unsalvageable was imaged after 28 weeks.

### Discrepancy between ultrasound and menstrual dates

Ultrasound confirmed the menstrual gestational age in 62.1% (110/177) of subjects. Thirteen percent (23/177) of the enrolled pregnant women were unaware of their menstrual dates and were therefore dated by the ultrasound. An additional 24.9% (44/177) of the subjects were re-dated by the ultrasound. Overall, ultrasound was useful in improving the accuracy of gestational age in over one third of the subjects.

### Delivery information

Of the 177 subjects with ultrasound confirmed dates, information about the delivery was missing for 23; twelve subjects withdrew from the trial, three relocated prior to delivery, seven were lost to follow-up, and ineligibility to the parent trial was later confirmed for one. Of the remaining 154 subjects, one delivered at home where neither a birth weight nor Ballard examination was performed. There were two stillbirths with recorded weights (Ballard examination not applicable). There were an additional four subjects with missing birth weights.

### Comparison of ultrasound with postnatal Ballard estimation

**Figure 4:**
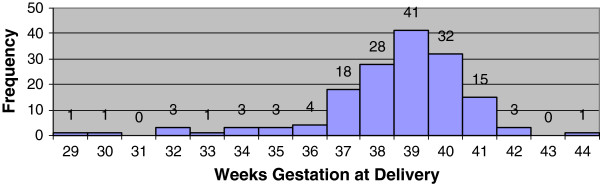
Distribution of gestational age in weeks at delivery by best obstetric estimate (ultrasound confirmed menstrual dates).

**Figure 5:**
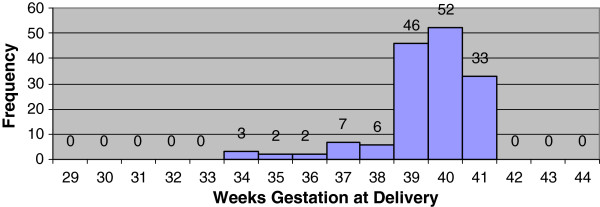
Distribution of gestational age in weeks at delivery by postnatal Ballard examination.

**Figure 6:**
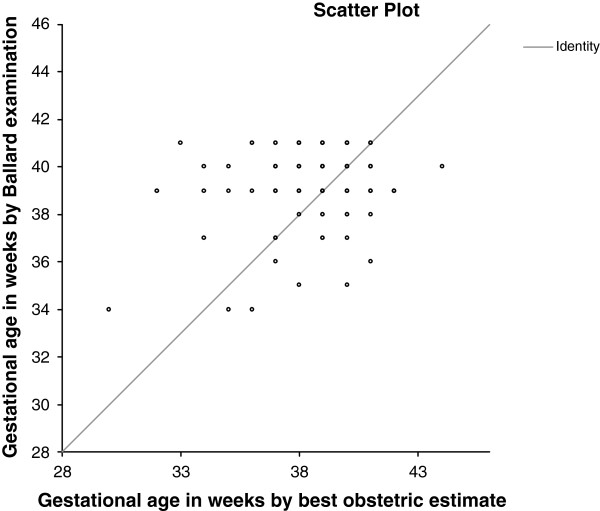
**Scatterplot of gestational age in weeks at delivery by Ballard examination **(**y**-**axis**) **versus by best obstetric estimate (x**-**axis). **Line of identity represents the plot if gestational age was the same for both methods for each subject.

**Figure 7:**
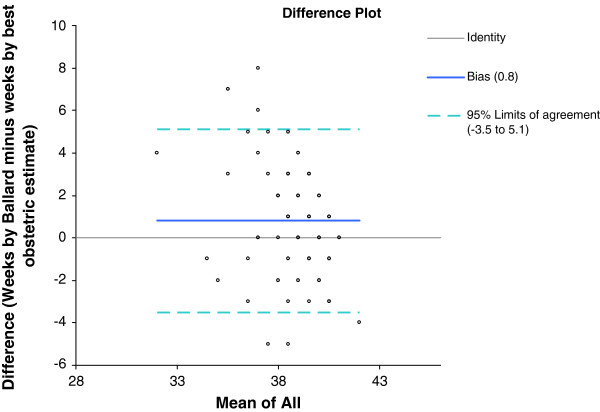
**Bland**-**Altman plot comparing agreement between two methods of gestational age assessment**, **Ballard examination and best obstetric estimate. **The difference between the two methods (Ballard minus best obstetric estimate) is plotted on the y-axis against the mean of the two methods on the x-axis. The identity line at y=0 represents values where the two methods yielded the same estimate of gestational age. The bias (the average difference between the two methods) is plotted as a solid line and the 95% limits of agreement represented by the dashed lines.

The distribution of gestational age at the time of delivery generated from the best obstetric estimate (ultrasound confirmed menstrual dates) and generated from Ballard examinations were compared (Figures 4 and 5). The median gestational age at delivery was 39 weeks for best obstetric estimate, range 29 to 44 weeks, and 39 weeks for the Ballard exam, range 34 to 41 weeks. The median gestational age differed significantly between the two methods (p <0.001). There was also very poor agreement between the two methods (Figures 6 and 7). On average, the bias of the Ballard examination was 0.8 weeks greater than the best obstetric estimate (95% confidence interval of 0.5 to 1.2). The lower limit of agreement was 3.5 weeks earlier (Ballard minus best obstetric estimate) with a 95% confidence interval for the lower limit of -4.1 to -2.9 weeks. The upper limit of agreement was 5.1 weeks later (Ballard minus best obstetric estimate) with a 95% confidence interval for the upper limit of 4.5 to 5.7. The estimates of bias and the limits of agreement did not significantly change after stratifying by whether the dating ultrasound occurred before or after 20 weeks’ gestation.

#### Misclassification of prematurity

Further comparison of the two histograms reveals a striking difference (Figures 4 and 5). While both distributions are right-skewed with the central tendency focused around term deliveries, the histogram of Ballard examinations appears to be clustered around the mean. Eighty seven percent (131/151) of the values fall between 39 to 41 weeks for Ballard examinations compared with only 57% (88/154) of the values estimated ultrasound confirmed menstrual dates (best obstetric estimate). Using the best obstetric estimate as the gold standard for gestational age, only 2.9% (4/137) were misclassified by the Ballard as preterm. However, 78.5% (11/14) of the live-born infants deemed to be preterm were misclassified as term by the Ballard examination suggesting an overestimation of gestational age. These misclassifications were then compared by the timing of the dating ultrasound. Ten of twelve infants (83.3%) identified as preterm by the best obstetric estimate and whose ultrasound scan occurred at 20 weeks or beyond were misclassified as term by the Ballard. For infants with an ultrasound prior to 20 weeks, 1 of 2 infants (50%) classified as preterm by the best obstetric estimate were misclassified as term by the Ballard.

#### Misclassification as small-for gestational age

Using the best obstetric estimate as the standard for the gestational age at the time of delivery, 27.8% (41/147) of infants with complete delivery information were considered small-for-gestational age using the reference curve generated for an East African population [28]. Using the Ballard to date the pregnancy, 11 of these 41 infants, or 26.8%, would be misclassified as average for gestational age. Additionally, the Ballard would misclassify 23.6% (25/106) average-for-gestational age infants as small-for-gestational-age.

## Discussion

This study demonstrated the feasibility of training midlevel non-obstetric providers in an African research setting to perform high quality ultrasound assessments of gestational age as have other research groups in similar resource-limited settings outside of Africa [29,30]. Over 94% of the image pairs for all biometric parameters were of acceptable quality. In only one subject were the authors unable to generate an ultrasound prediction of the date of delivery. Nonetheless, it should be emphasized that the training was time intensive and that there was a considerable learning curve. Research staff did not become skilled in biometric imaging after the first week-long course. The four months of additional remote image feedback were instrumental in achieving the quality required in the formal demonstration pilot. In addition, ongoing review of unused images provided an opportunity for the research staff performing ultrasound to maintain and improve their skills. Rejected images were notably more frequent at the beginning of the study period.

In addition to feasibility, it was of demonstrable utility to incorporate ultrasound into gestational age assessment compared with reliance on menstrual dates alone. For over one third of the subjects, ultrasound scans were used to improve the accuracy of their estimated date of delivery. It is possible that the results overstate the utility given that not all subjects in the parent trial underwent ultrasound examination. For women with unknown dates, there may have been added motivation on the part of research staff and/or subjects to enroll a woman. Of note, no woman refused ultrasound examination and anecdotally appeared pleased with the procedure.

Ultrasound could be used to verify gestational age at the time of enrollment into a research study involving pregnant women, screening out those women whose pregnancies are too far advanced to meet inclusion criteria. In addition to improving gestational age assessment, other benefits would likely accrue to pregnancy cohorts if ultrasound were incorporated as part of enrollment procedures. These include the ability to confirm a live pregnancy, an intrauterine location, and whether the pregnancy was a multiple gestation.

A striking finding of this study was the comparison of postnatal gestational age assessment by Ballard examination with antenatal assessment using the protocol for ultrasound confirmation of menstrual dates, referred to throughout this manuscript as the best obstetric estimate of gestational age. There was extremely poor agreement between the Ballard and the best obstetric estimate as evidenced in the Bland-Altman plot. How is one to know which represents the ‘true’ or gold-standard gestational age? The histograms of gestational age distribution between these two methods may help clarify the answer. The gestational age by Ballard examinations are all clumped around 39 to 41 weeks with very few deliveries deemed by Ballard to be preterm or even early term (37 to 38 weeks). The Ballard examination, as practiced in a real world African research setting in this study, yielded values that do not appear dispersed enough. Only 4.6% of infants were characterized as being born preterm by Ballard compared with approximately 10% by the best obstetric estimate, the latter figure more in line with preterm delivery rates reported by the World Health Organization for sub-Saharan African [31].

The tendency of the Ballard in this study to characterize most deliveries as term likely led to misclassification. In fact, almost 80% of the infants identified as premature by the best obstetric estimated were classified by Ballard as term. As only a minority of the subjects’ ultrasound scans occurred prior to 20 weeks, it is possible that the best obstetric estimate incorporating ultrasound measurements underestimated gestational age in cases of growth restriction, inappropriately deeming such growth-restricted infants preterm. Evidence of foetal growth restriction following malaria infection has been demonstrated as early as the second trimester [13]. Nonetheless, half of the subjects scanned before 20 weeks and classified as having a preterm birth by the best obstetric estimate also were considered term deliveries by Ballard examination. The numbers are too small for definitive conclusions but suggest that not all of the potential misclassification can be attributed growth restriction missed by ultrasound dating. The World Health Organization estimates the preterm birth rate for Malawi to be 18 percent [31]. In the study, the proportion of preterm births by the best obstetric estimate was much lower, at 10.3% (16/154). It is, therefore, possible that our ultrasound protocol also underestimated gestational age.

Since the original publication of the new Ballard score in 1991, not all studies have been able to reproduce its validity. Several suggest, as did the present one, that the Ballard score overestimated the gestational age in preterm infants leading to an underestimation of the percentages of preterm infants [32,33]. A study from Brazil reported poor sensitivity (70%) in detecting preterm infants when compared with ultrasonography [34]. In one study from Malawi, the external features of the Ballard were reliably obtained by nurses and led to an improvement in gestational age assessment when compared with dating by the last menstrual period or the fundal height [35]. No comparison, however, was made with ultrasound. The present study suggests a potential improvement in accuracy incorporating ultrasound into gestational age assessment. However, it is quite likely that an intense four month period of training in the Ballard examination and ongoing quality control of all examinations would have improved the reliability of the Ballard examination. With postnatal gestational age assessment, the number of items used to score the newborn is associated with the accuracy yet most scoring methods have reduced the number of items for simplicity and practicality [36,37]. This underscores the need for appropriate training and quality control in these assessments.

Why not simply date the pregnancy by ultrasound alone and eliminate the somewhat cumbersome process of determining the best obstetric estimate of the EDD? The earlier in pregnancy ultrasound is used, the more accurate the estimation of gestational age. Foetuses are of similar size early in pregnancy. Variation in size increases with advancing gestational age as individual genetics, nutrition, infections or other exposures may impact how a foetus grows. First trimester ultrasound is the most accurate [38]; however, in many resource-limited settings women do not seek prenatal care this early and may not be identified during the first trimester for participation in pregnancy cohorts. Dating by ultrasound alone may be valid for first trimester ultrasound and even up to 20 weeks when the discrepancy with ‘true dates’ is a week or less. However, when measurement error approaches +/- two weeks, the combination of ultrasound with menstrual dates appears to be a preferable strategy. This ‘best estimate’ approach is used clinically by most obstetricians in practice.

Alternative gestational age assessment will still be required for the many pregnant women in Africa, even those recruited to research studies, who present in the latter half of pregnancy for establishment of care. The limitations of ultrasound biometry late in gestation must not be overlooked. Pooled data combining ultrasound measurements with other gestational age data from menstrual dates, symphysis-fundal heights, and/or postnatal exams should be considered [39]. When ultrasound is available, measurements of the transcerebellar diameter are more accurate in establishing gestational age late in gestation when compared with measurements of the head, abdomen, and leg as cerebellar size appears independent of abnormalities of foetal growth (growth restriction or overgrowth) [40]. Nomograms exist correlating transcerebellar diameter with gestational age and appear to accurately predict gestational age even in the late second and third trimesters [41,42]. Whether such measurements could be obtained in resource-limited settings with non-obstetric providers should be evaluated prospectively.

There are multiple limitations to this study. It was envisioned as a demonstration project to verify the ability to train non-obstetric providers in quality image acquisition of ultrasound biometry measurements. While the images obtained were remotely determined to be of quality or not by the study perinatologist using predetermined standard criteria, there was no formal assessment of inter-observer reliability as has been done by other groups [29,30]. Gestational age measurements could have been compared between the individual non-obstetric providers in Malawi or between the non-obstetric providers and the study perinatologist. Intra-observer reliability was also not formally assessed. Secondly, the comparison of ultrasound with the postnatal Ballard could be criticized for lack of a similarly rigorous quality control for the Ballard exams or for inclusion of subjects whose ultrasound measurements occurred during the third trimester. Nonetheless, such shortcomings do highlight the challenge of gestational age assessment even in research settings.

In summary, this study demonstrates that the introduction of ultrasound for antenatal confirmation of gestational age in a malaria pregnancy cohort is technically feasible even in the hands of non-obstetric research staff supported with appropriate training and feedback. It is probable the addition of ultrasound is less likely to lead to gestational age misclassification when compared with postnatal Ballard. Gestational age misclassifications in malaria pregnancy studies could yield inaccurate conclusions about the effects of antenatal malaria infection. The classical teaching is that in areas where malaria is endemic, malaria infection leads to growth restriction but that preterm labour is not a frequent occurrence [1]. In contrast, in areas where malarial transmission is unstable, pregnancy malaria can trigger preterm labour. If the Ballard examination systematically overestimates gestational age, perhaps premature delivery has been underappreciated as a consequence of antenatal malaria. The classical teachings should be verified in cohorts with well-dated pregnancies.

The implications of accurate gestational age assessment extend well beyond malaria pregnancy studies as the knowledge of valid pregnancy dates is required for appropriate obstetric care. The present work suggests that midlevel non-obstetric providers can be trained to provide this information. Training a broad cadre of mid-levels to perform ultrasound biometry has the potential not only to strengthen research endeavours but also to improve obstetric care in resource-limited settings. Yet, inclusion of ultrasound in gestational age assessment is more than simply providing a machine and keeping it serviced. It requires investments of time in both initial training and ongoing continuing quality control underscoring the need for strong partnerships between settings where expertise in such skills does and does not exist.

## Conclusions

Midlevel non-obstetric providers can be trained to perform high quality ultrasound scans for foetal biometry with a period of intense training and subsequent ongoing review. There was poor agreement between the ultrasound confirmed gestational age and postnatal Ballard examination. The distribution of gestational age by Ballard examination is atypical and suggests overestimation of preterm infants as term. When compared with the best obstetric estimate combining menstrual dates with an ultrasound protocol, Ballard exam misclassified approximately 80% of preterm infants as term. Ultrasound, with appropriate training and quality control, should be strongly considered in prospective malaria studies with obstetric endpoints to avoid misclassification of gestational age and/or growth.

## Abbreviations

AC: Abdominal circumference; BPD: Biparietal diameter; EDD: Estimated date of delivery; FL: femur length; U/S: Ultrasound.

## Competing interests

No authors have financial or non-financial competing interests to declare.

## Authors’ contributions

BJW and MKL conceived of and designed the study protocol. BJW performed ultrasound training and oversight. PM, ON, RK, AM, PT, GM, LKP, MM, and BM were responsible for the conduct and oversight of all study procedures. BJW and AM contributed to data analysis. BJW and MKL were the primary contributors to manuscript preparation with editing and approval by the remaining authors. All authors read and approved the final manuscript

## Supplementary Material

Additional file 1 Standard operating procedure for the new Ballard examination. Click here for file
