# Supplementary material for: Gestational age assessment in malaria pregnancy cohorts: a prospective ultrasound demonstration project in Malawi
Source: Malar J. 2013 Jun 4;12:183. doi: 10.1186/1475-2875-12-183 (PMC3679840; doi:10.1186/1475-2875-12-183)
Supplement: Additional file 1 — Standard operating procedure for the new Ballard examination. [file 1475-2875-12-183-S1.doc]

APPENDIX 1: Standard operating procedure for the new Ballard examination

Reference: Ballard JL, Khoury JC, Wedig K, Wang L, Eilers-Walsman BL, Lipp R: **New Ballard Score, expanded to include extremely premature infants**. *J Pediatr* 1991, **119**:417-23.

The new Ballard scoring method uses both 1) neurological features and 2) external features. Each feature is given a score and these scores are added up to give a final score. This final score can be converted to an estimated gestational age—see Table at the end of this appendix.

Neurological features of the Ballard score

All 6 neurological features are assessed with the infant lying on its back (back to the bed). The infant should be awake but not crying.

POSTURE: Handle the infant and observe the position of the arms and legs. More mature infants (with a higher gestational age) have better flexion (tone) of their limbs.

Score 0 if both arms and legs are fully extended

Score 1 if there is slight flexion of the legs only.

Score 2 if there is moderate flexion of the legs.

Score 3 if the legs are flexed to 90 degrees and the arms are partially flexed.

Score 4 if all limbs are fully flexed against the body.

SQUARE WINDOW: Gently press on the back of the infant’s hand to push the palm towards the forearm. Observe the degree of flexion. More mature infants have greater wrist flexion.

Score -1 if the wrist cannot even be flexed to 90 degrees

Score 0 if the wrist can only be flexed to 90 degrees, giving the appearance of a ‘square window.’

Score 1 if the wrist can be flexed to 60 degrees.

Score 2 if the wrist can be flexed half way to the forearm.

Score 3 if the wrist can be flexed to 30 degrees.

Score 4 if the palm of the hand can be pressed against the arm.

ARM RECOIL: Fully bend the arm at the elbow so that the infant’s hand reaches the shoulder, and keep it flexed for 5 seconds. Then fully extend the arm by pulling on the fingers. Release the hand as soon as the arm is fully extended and observe the angle of flexion at the elbow (recoil). Arm recoil is better in more mature infants.

Score 0 if there is no arm recoil at all.

Score 1 if there is slight arm recoil- an angle at the elbow of only 140 to 160 degrees.

Score 2 if there is some arm recoil (110-140 degree angle).

Score 3 if the arm recoil is good and the arm is flexed approximately half way back to the shoulder (90-110 degrees).

Score 4 if there is brisk arm recoil and the infant pulls the arm back almost to the shoulder (less than 90 degrees).

POPLITEAL ANGLE: With your one hand, hold the infant’s knee against the abdomen. With the index finger of the other hand, gently push behind the infant’s ankle to bring the foot towards the face. Observe the angle formed behind the knee by the upper and lower legs (the popliteal angle). More mature infants have less extension of the knee.

Score -1 if the leg can by fully extended to form an angle of 180 degrees.

Score 0 if there is some limitation to the full extension of the leg (an angle of approximately 160 degrees).

Score 1 if there is some limitation to the full extension of the leg (an angle of approximately 140 degrees).

Score 2 if the knee can only be extended to 120 degrees.

Score 3 if the knee can be extended just beyond 100 degrees.

Score 4 if the knee can be extended only to 90 degrees.

Score 5 if the knee cannot be extended to 90 degrees.

SCARF SIGN: Take the infant’s hand and gently pull the arm across the front of the chest and around the neck like a scarf. With your other hand gently press on the infant’s elbow to help the arm around the neck. In more mature infants the arm cannot be easily pulled across the chest.

Score -1 if the arm can by wrapped tightly around the neck (like a scarf).

Score 0 if the elbow can only be pulled well across the chest but not fully wrapped around the neck.

Score 1 if the elbow reaches the other side of the chest but cannot be pulled beyond the chest.

Score 2 if the elbow reaches the midline of the chest but cannot be pulled all the way to the other side of the chest.

Score 3 if the elbow cannot reach the midline of the chest.

Score 4 if the elbow cannot be pulled across the chest at all.

HEEL TO EAR: Hold the infant’s toes and gently pull the foot towards the ear. Allow the knee to slide down at the side of the abdomen. Unlike the illustration, the infant’s pelvis may be allowed to lift off the bed. Observe how close the heel can be pulled towards the ear. More mature infants have less flexion of the hips, and, therefore, you cannot bring the heels towards the ear.

Score -1 if the heel can easily be pulled to the ear.

Score 0 if the heel almost reaches the ear.

Score 1 if the heel can be pulled most of the way to the ear.

Score 2 if the heel can be pulled beyond the halfway mark but not most of the way to the ear.

Score 3 if the heel can be pulled half way to the ear.

Score 4 if the heel cannot be pulled halfway to the ear.

External features of the Ballard score

Six external features are examined. The infant has to be turned over to examine the amount of lanugo. If the infant is too sick to be turned over, then the amount of lanugo is not scored.

SKIN: Examine the skin over the front of the chest and abdomen, and also look at the limbs. More mature infants have thicker skins.

Score -1 if the skin appears sticky, friable and transparent.

Score 0 if the skin appears very thin, red, translucent and gelatinous (jelly-like).

Score 1 if the skin is thin and smooth with many small blood vessels visible.

Score 2 if the skin is thicker with only a few blood vessels seen. Fine peeling of the skin is often noticed, especially around the ankles.

Score 3 if the skin is pale and slightly dry or cracking with only a few bigger blood vessels seen.

Score 4 if the skin is dry and deeply cracked with no blood vessels visible.

Score 5 if the skin is very thick and looks like leather.

LANUGO: This is the fine, fluffy hair that is seen over the back of small infants. Except for very immature infants that have no lanugo, the amount of lanugo decreases with maturity.

Score -1 if no lanugo is seen. These are very small infants.

Score 0 if lanugo is sparse. These are very small infants.

Score 1 if the lanugo is thick and present over most of the back.

Score 2 if the lanugo is thinning, especially over the lower back.

Score 3 if there are bald areas with no lanugo.

Score 4 if very little lanugo is seen. These are always bigger infants.

PLANTAR SURFACE: Use your thumbs to stretch the skin on the bottom of the infant’s foot. Only note creases. Very fine wrinkles, that disappear with stretching, are not important and do not count as creases. More mature infants have more creases.

Score -2 if the heel-toe measurement if less than 40 mm.

Score -1 if the heel-toe measurement is between 40 and 50 mm.

Score 0 if there are no creases at all (there may be fine wrinkles) and the heel-toe measurement is greater than 50 mm.

Score 1 if shallow, faint red creases are present, especially over the anterior sole.

Score 2 if deeper creases are present on the anterior third of the sole only.

Score 3 if deep creases are present over two thirds of the sole.

Score 4 if the whole sole is covered with deep creases.

BREAST: Both the appearance of the breast and the size of the breast bud are considered. Palpate for the breast bud by gently feeling under the nipple with your index finger and thumb. More mature infant have a bigger areola and breast bud.

Score -1 if the breast is imperceptible.

Score 0 if the areola (pink skin around the nipples) is very small and difficult to see (barely perceptible).

Score 1 if the areola is small and flat, and no breast bud can be felt.

Score 2 if the breast bud can just be felt and the areola is stippled (has fine bumps).

Score 3 if the areola is raised above the surrounding skin and the breast bud is

easily felt (3-4 mm).

Score 4 if the areola appears distended and the breast bud is the size of a pea (5- 10mm).

EYE/EAR: Both the degree of eyelid fusion and the shape and thickness of the external ear are considered. With increasing maturity, the edge of the ear curls in. In addition, the cartilage in the ear thickens with maturity so that the ear springs back into the normal position after it is folded against the infant’s head.

Score -2 if the eyelids are tightly fused.

Score -1 if the eyelids are loosely fused.

Score 0 if the eyelids are open ear is soft and flat and stays folded.

Score 1 if the eyelids are open and the ear slowly unfolds. The upper margin of the ear (pinna) has started to curl in.

Score 2 if the upper margin of the ear is well curled and the ear unfolds quickly. Areas of cartilage still feel soft, especially towards the edge of the ear.

Score 3 if the cartilage feels firm throughout the ear, and the ear springs back rapidly if folded.

Score 4 if the ear feels stiff and the whole ear margin is well curled in.

GENITALIA: Male and female genitalia are scored differently. With maturity, the testes descend in the male and the scrotum becomes wrinkled. In females, the labia majora increases in size with maturity.

MALES:

Score -1 if the scrotum is flat and smooth.

Score 0 if the scrotum is very small but no longer flat and smooth with no testes palpable.

Score 2 if there are a few wrinkles (rugae) in the scrotum and one or both testes are felt in the upper canal.

Score 3 if the testes are in the scrotum and the skin of the scrotum has a lot of wrinkles.

Score 4 if the scrotum hangs low with fully descended testes.

FEMALES:

Score -1 if the clitoris is prominent and the labia majora are flat and not formed.

Score 0 if the labia majora (outer labia) are only slightly formed, still leaving the labia minora (inner labia) and clitoris completely exposed.

Score 2 if the labia majora and labia minora are of equal size.

Score 3 if the labia majora are bigger than the labia minora.

Score 4 if the labia majora cover the clitoris and labia.

| ***Maturity Rating -- Ballard Method*** | |
| --- | --- |
| **Total Score^†^** | **Gestational age (Weeks)** |
| -10 | 20 |
| -5 | 22 |
| 0 | 24 |
| 5 | 26 |
| 10 | 28 |
| 15 | 30 |
| 20 | 32 |
| 25 | 34 |
| 30 | 36 |
| 35 | 38 |
| 40 | 40 |
| 45 | 42 |
| **50** | 44 |

**^†^**For scores that fell between the 5-point increments, we extrapolated using a method recommended by Ballard. For example, if a score of 15 corresponded to 30 weeks gestation and a score of 20 corresponded to 32 weeks; a score of 16 was assigned to 30 weeks, and the scores of 17, 18, and 19 were assigned 31 weeks.
